# Relationship between serum B12 concentrations and mortality: experience in NHANES

**DOI:** 10.1186/s12916-020-01771-y

**Published:** 2020-10-09

**Authors:** Bruce H. R. Wolffenbuttel, M. Rebecca Heiner-Fokkema, Ralph Green, Rijk O. B. Gans

**Affiliations:** 1 Department of Endocrinology, University of Groningen, University Medical Center Groningen, P.O. Box 30001, HPC AA31, 9700 RB Groningen, The Netherlands; 2 Department of Laboratory Medicine, University of Groningen, University Medical Center Groningen, Groningen, The Netherlands; 3 Department of Pathology and Laboratory Medicine, University of California Davis, Sacramento, CA USA; 4 Department of Internal Medicine, University of Groningen, University Medical Center Groningen, Groningen, The Netherlands

**Keywords:** Vitamin B12, Epidemiology, NHANES, Prognosis, Mortality, Supplement

## Abstract

**Background:**

There is conflicting evidence in the literature on the association between (elevated) serum B12 concentrations and subsequent disease or mortality. We evaluated in the NHANES general population the association of serum B12 concentrations as well as vitamin B12 supplement intake with all-cause, cardiovascular, and cancer-related mortality, while taking into account demographic and lifestyle factors and significant other diseases which are known to be associated with poorer outcome.

**Methods:**

The main outcomes of our study were all-cause mortality, cardiovascular mortality, and cancer-related mortality. Mortality status and cause of death were determined by NHANES-linked National Death Index public access files through December 31, 2015. The association of serum B12 concentrations and vitamin B12 supplement intake with mortality was assessed with Cox proportional hazard (PH) models, with adjustment for a number of relevant demographic and lifestyle factors and comorbidity.

**Results:**

The final study population of 24,262 participants had a mean age of 48 (SD 19) years; 50.1% were males. The median follow-up duration was 109 months (range 1–201 months). On the census day of December 31, 2015, 3023 participants were determined as deceased (12.5%). The fully adjusted Cox PH model indicated that low serum B12 concentrations < 140 pmol/l were associated with a small increase in all-cause (hazard ratio, HR 1.39, 95% CI 1.08–1.78, *p* = 0.011) and cardiovascular (HR 1.64, 95% CI 1.08–2.47, *p* = 0.020) mortality. Similarly, high serum B12 concentrations > 700 pmol/l were associated with an increase in cardiovascular mortality only (HR 1.45, 95% CI 1.01–2.06, *p* = 0.042). Participants with a diagnosis of hypertension, dyslipidemia, CVD, and cancer more frequently used vitamin B12-containing supplements than those without these diagnoses. We could not demonstrate an association between vitamin B12 supplement intake and mortality, when adjusted for comorbidity.

**Conclusions:**

In the general population of NHANES, low serum B12 concentrations were associated with a moderate increase in all-cause mortality. There was a small but significant increase in cardiovascular mortality in the groups with low or high serum B12. High intake of vitamin B12 in the form of supplements was not associated with any adverse effect on mortality and therefore can be regarded as safe.

## Background

Vitamin B12 is an essential nutrient which has an important role in many processes in the body, including DNA synthesis and DNA methylation, synthesis of blood cells, and nerve function. Metabolically, vitamin B12 is closely associated with folate in the regulation of key elements involving the transfer of one-carbon units [1]. Animal products like meat, poultry, fish, eggs, and milk are the exclusive dietary sources for vitamin B12 in human nutrition [2]. The uptake of vitamin B12 in the body is rather complex, and both salivary production of certain B12-binding proteins, intrinsic factor in the stomach, gastric acid, and pancreatic enzymes play a role in absorption which ultimately takes places in the ileum [3]. Deficiency may occur either when the food does not contain sufficient animal products or when there is interference in the uptake and absorptive mechanisms, although several other factors, including exposure to nitrous oxide, may be involved [1, 4–6].

Excess intake of vitamin B12 or treatment of vitamin B12 deficiency with intramuscular injections has been considered safe for over half a century, as the compound is water-soluble. Any excess of an injected dose will be excreted in the urine, and the ability to absorb excessive quantities of orally administered B12 is limited by the restricted capacity of the receptors in the ileum [1]. Nevertheless, there have been several reports on the association between serum B12 concentrations and increased risk of disease, i.e., risk of lung cancer [7], or even mortality. Although most epidemiologic studies showed no association between serum B12 and cardiovascular disease [8–12], a recent paper suggested that higher serum B12 concentrations were associated with increased risk of mortality [13]. However, it should be noted that a causal relationship between serum B12 concentrations and subsequent disease or mortality has never been demonstrated. Increased serum B12 concentrations may be a proxy for increased intake of red meat, inflammation due to other chronic diseases, or intake of supplements as a complementary therapy for other conditions. One very recent paper using data from the Women’s Health Initiative (WHI) study in postmenopausal women showed no association between vitamin B12 (supplement) intake and risk of lung cancer [14].

The National Health and Nutrition Examination Survey (NHANES), a long-term epidemiologic survey in the USA [15], has been the source for several papers on B12 and related disorders. Serum B12 concentrations have been measured in several NHANES surveys. Recently, we demonstrated in NHANES that serum methylmalonic acid (MMA) was more strongly associated with impairments of cognitive and physical functioning than serum B12 concentration [16]. Because of the high-level survey quality and the availability of mortality data, we aimed to evaluate in NHANES the association between serum B12 concentrations as well as vitamin B12 supplement intake and all-cause, cardiovascular, and cancer-related mortality. In the analyses, we adjusted for significant demographic and lifestyle factors and comorbidities which are known to be associated with poorer outcomes.

## Methods

### NHANES structure and inclusions

NHANES is a cross-sectional survey in the USA that uses a complex, stratified, multistage probability sampling design [15, 17]. The survey protocol was approved by the Research Ethics Review Board of the National Center for Health Statistics, and NHANES has obtained written informed consent from all participants. For this analysis, we used publicly available data without personal identifiable information, and all methods were carried out in accordance with relevant guidelines and regulations. Interview questionnaires and examination response rates are publicly available [18]. Participants were first interviewed in their homes, during which demographic information and a variety of health-related information were collected. One to 2 weeks later, subjects underwent a standardized physical examination, as well as additional investigations like exercise testing, 24-h dietary recall, and a blood draw in a mobile examination center. Blood samples were taken with the participant fasting. Participants who visited the examination in the morning were requested to fast for 9 h, those visiting in the afternoon or evening were requested to fast for 6 h. For this study, we included all adults aged 18 years or older who participated in the continuous NHANES survey cycles of 1999–2000 through 2013–2014, in whom serum B12 concentrations were assessed at baseline (Additional file 1: Fig. 1). A total of 82,091 people participated in the 1999–2000 through 2013–2014 NHANES surveys. Of those, 47,279 participants were eligible for mortality follow-up. We excluded 974 women who reported to be pregnant or breastfeeding. Serum B12 was measured in 6 of the 8 2-year surveys and available in 24,262 non-pregnant participants (51.3%).

**Fig. 1:**
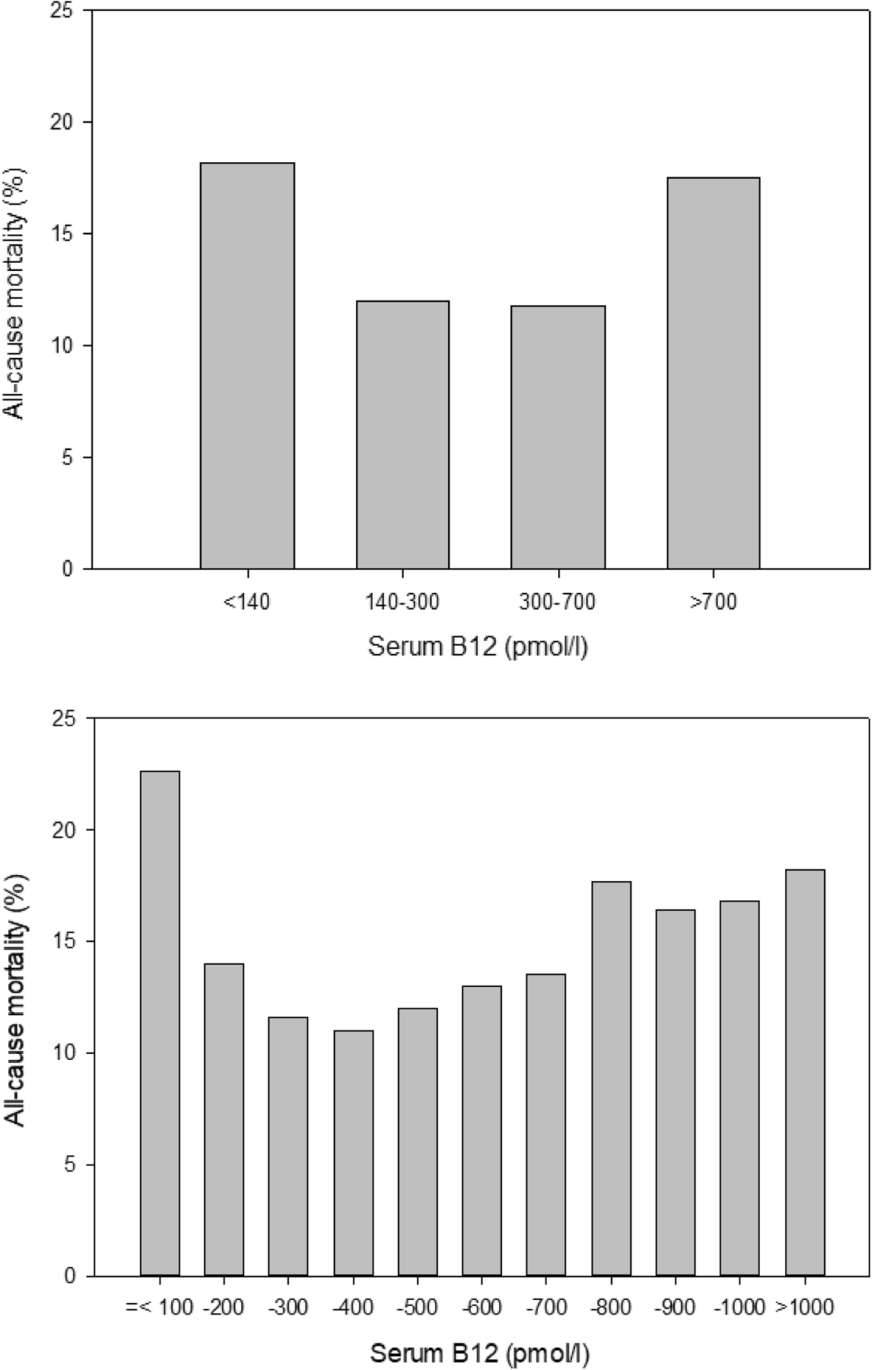
Unadjusted all-cause mortality in NHANES 1999–2014 according to serum B12 concentrations

### Measurements

NHANES blood samples were processed, stored, and shipped to the Collaborative Laboratory Services, Ottumwa, IA. For the 1999 through 2006 surveys, serum B12 and folate concentrations (*n* = 14,194) were measured by using the Bio-Rad Laboratories “Quantaphase II Folate/vitamin B12” radioassay kit (Bio-Rad Laboratories, 1993). In 2011–2014, serum B12 (*n* = 10,068) was measured using the fully automated Roche electrochemiluminescence immunoassay on a Modular Analytics E170® system (Roche Diagnostics, Indianapolis, IN). The two methods have similar precision (< 5% CV) and limits of detection (LOD): 20 pg/ml for the Bio-Rad assay and 30 pg/ml for the Roche assay. Because Deming regression considered errors in both variables, we converted all concentrations to the BioRad values, with the proposed equation serum B12 Bio-Rad in pg/ml = 10^(1.03 × log10(Roche vitamin B12) − 0.14). This re-calculation was also based on the literature evaluating several serum B12 assays, in which the Elecsys system yielded results significantly higher than the international standard [19]. In the 2013–2014 survey, NHANES quality control showed significant shifts associated with reagent lot-to-lot variations and adjusted the serum B12 measurements using mean quality control pool data using a Deming regression [20]. In the 2011–2014 surveys, separate folate forms were measured with isotope-dilution high-performance liquid chromatography coupled to tandem mass spectrometry (LC-MS/MS), and serum total folate was calculated by the addition of the separate folate forms. The folate values of the 1999–2006 surveys were re-calculated according to NHANES instructions. Serum creatinine was measured with the Jaffé rate method (kinetic alkaline picrate) on a Beckman Synchron DxC800 modular chemistry analyzer. Renal function was calculated as an estimated glomerular filtration rate (eGFR) with the formula developed by the Chronic Kidney Disease Epidemiology Collaboration (CKD-EPI) [21]. Hemoglobin measurements and white blood cell count (WBC) were performed with a Beckman Coulter MAXM between 1999 and 2012 and the Beckman Coulter DxH 800 in 2013–2014 (Beckman Coulter Inc., Brea, CA, USA). No significant trend changes for hemoglobin was reported from NHANES 1999–2012 to NHANES 2013–2014, but there was a small change in weighted mean WBC, from 7.03 × 10^3^ cells/μl in 2011–2012 to 7.41 × 10^3^cells/μl in 2013–2014. As we used WBC only as a covariate in our regression models, we judged that this small change did not warrant recalculation of the results. C-reactive protein was measured with latex-enhanced nephelometry on a Behring nephelometer (1999–2010), serum cholesterol was measured with an enzymatic method on a Hitachi 704 Analyzer (Roche Diagnostics), plasma glucose with a hexokinase method on a Cobas Mira system, and HbA1c with HPLC methodology (1999–2004 Primus CLC330 and Primus CLC 385 (Primus Corporation, Kansas City, MO), 2005–2006 on a Tosoh A1c 2.2 Plus Glycohemoglobin Analyzer, from 2007 onwards A1c G7/G8 HPLC Glycohemoglobin Analyzer (both Tosoh Medics, Inc., San Francisco, Ca). Plasma total homocysteine (HCys) was measured in 1999–2006 by using a fluorescence polarization immunoassay (Abbott Laboratories) [22]. Serum MMA concentrations were analyzed by GC/MS (1999–2004) and LC-MS/MS (2011–2014). Both methods had an excellent correlation and no bias [23]. All information regarding these methods is publicly available on the NHANES website [17, 24].

Medication use was scored in NHANES by the unique generic drug code from Multum’s Lexicon Drug Database. The number of different medications reported by a participant was considered as a proxy for comorbidity [25]. The NHANES Dietary Supplements section provides personal interview data on the use of vitamins, minerals, and other dietary supplements. In the surveys 1999 through 2006, daily vitamin B12 intake was calculated by combining the daily dose of any supplement with its use per month. For all supplements, reference concentrations are given in the Dietary Supplement Database. From these, we calculated the average daily intake of vitamin B12 as a supplement. Some participants did not report frequency or dosing information or reported fewer than seven dosings per month. In these, we considered the daily vitamin B12 supplement intake as unreliable, and therefore, it was set as “missing.” In the surveys 2007 through 2014, total supplement intake has already been reported for a number of vitamins and minerals, including vitamin B12, in the total dietary supplements tables.

### Outcomes

The main outcomes of our study were all-cause mortality, cardiovascular mortality, and cancer-related mortality. Mortality status and cause of death were determined by NHANES-linked National Death Index public access files through December 31, 2015.

### Statistical analysis

Our study population was categorized by specific serum B12 groups: (1) group with low serum B12 concentrations, defined as a serum B12 concentration < 140 pmol/l; (2) “possible deficiency,” serum B12 concentrations between 140 and 300 pmol/l; (3) normal concentrations, serum B12 between 300 and 700 pmol/l; and (4) elevated serum B12 concentrations > 700 pmol/l. Daily oral intake of vitamin B12-containing supplements was categorized as (1) no supplement intake, (2) 0.1–4.9 mcg, (3) 5.0–24.9 mcg, (4) 25.0–99 mcg, (5) 100–999 mcg, and (6) ≥ 1000 mcg intake.

The association of serum B12 concentrations and vitamin B12 supplement intake with all-cause mortality, cardiovascular mortality, and cancer mortality was assessed with Cox proportional hazards (PH) analysis. The group with serum B12 between 300 and 700 pmol/l was set as the reference group. The confounders which were used in the models, were demographic factors, socioeconomic and lifestyle factors, and relevant comorbidities and laboratory measurements (for details see Additional file 2, Table 1). We plotted cumulative Kaplan-Meier curves for mortality during follow-up according to the predefined groups of serum B12 concentrations with the Stata STKAP module [26]. Cox PH regressions were used to estimate hazard ratios (HRs) and 95% CI for associations between serum B12 and mortality. We built four models to provide statistical inference. Model 1 only comprised serum B12 concentrations, model 2 included demographic variables (age, gender, ethnicity), and model 3 included variables from model 2 plus socioeconomic and lifestyle variables. Finally, model 4 adjusted for all variables (Additional file 2, Table 1), with the application of the specific sampling weights according to the NHANES’s instructions [27–29]. As a sensitivity analysis, we recalculated the Cox PH model for all-cause mortality in only participants of the 1999–2006 surveys (without and with the inclusion of homocysteine concentrations and C-reactive protein as an additional adjustment for chronic inflammation) and separately only for those participants in whom also serum concentrations of MMA were available.

**Table 1:** Baseline characteristics of participants who were still alive vs those who were deceased by December 31, 2015

		Still alive, ***N*** = 21,239	Deceased, ***N*** = 3023	***p*** value
Gender, M/F (*n*)	24,262	10,449/10,790	1712/1311	2.0 × 10^−14^
Age 18–39 years (%)	24,262	41.7	6.0	< 1.0 × 10^−200^
Age 40–59 years (%)		32.9	14.3	
Age ≥ 60 years (%)		25.4	79.7	
Body mass index (kg/m^2^)	23,920	28.6 ± 6.7	28.0 ± 6.2	8.8 × 10^−7^
Waist circumference (cm)	23,245	97 ± 16	100 ± 15	2.0 × 10^−18^
SBP (mmHg)	23,298	123 ± 18	138 ± 25	< 1.0 × 10^−200^
DBP (mmHg)	23,118	71 ± 11	69 ± 13	2.4 × 10^−17^
WBC (× 10^9^/l)	24,235	7.2 ± 2.2	7.4 ± 3.3	2.1 × 10^−10^
Hemoglobin (mg/dl)	24,235	14.2 ± 1.5	14.0 ± 1.7	1.5 × 10^−15^
C-reactive protein (mg/dl)	14,174	0.19 (0.07–0.44)	0.29 (0.14–0.67)	3.9 × 10^−70^
Cholesterol (mmol/l)	24,138	5.0 ± 1.1	5.1 ± 1.2	2.5 × 10^−7^
Glucose (mmol/l)	24,141	5.5 ± 2.0	6.2 ± 2.8	1.8 × 10^−63^
HbA1c (%)	24,225	5.6 ± 1.0	6.0 ± 1.3	9.7 × 10^−77^
Creatinine (μmol/l)	24,141	72 (62–88)	80 (71–106)	8.7 × 10^−80^
eGFR (ml/min/1.73 m^2^)	24,141	100 (84–116)	77 (56–94)	< 1.0 × 10^−200^
Serum B12 (pmol/l)	24,262	343 (257–463)	354 (252–494)	0.016
Serum folate (nmol/l)	24,182	38.7 (27.4–53.4)	37.7 (32.7–69.9)	8.4 × 10^−89^
Methylmalonic acid (nmol/l)^1^	19,492	130 (100–172)	175 (126–252)	4.9 × 10^−158^
Homocysteine (μmol/l)^2^	13,681	7.7 (6.4–9.3)	10.4 (8.3–13.3)	< 1.0 × 10^−200^
Ethnicity	24,262			3.5 × 10^−71^
Mexican American (*n*)		3927	568	
Other Hispanic (*n*)		1433	90	
Non-Hispanic White (*n*)		9077	1730	
Non-Hispanic Black (*n*)		4811	543	
Others, including multi-racial (*n*)		1991	92	
Education	22,730			1.8 × 10^−166^
Level 1 (%)		10.4	25.2	
Level 2 (%)		14.4	19.7	
Level 3 (%)		22.9	23.4	
Level 4 (%)		29.5	20.2	
Level 5 (%)		22.8	11.5	
Annual family income	22,348			1.9 × 10^−110^
< 25k (%)		36.3	56.0	
25–75k (%)		41.3	36.5	
> 75k (%)		22.4	7.5	
Never smoking (%)	23,179	51.7	39.9	3.0 × 10^−33^
Former smoking (%)	24,262	19.4	35.5	7.8 × 10^−90^
Current smoking (%)	24,262	26.5	24.0	0.003
Diabetes diagnosis (%)	24,262	14.1	29.0	1.0 × 10^−97^
Hypertension diagnosis (%)	24,262	30.6	60.3	2.4 × 10^−226^
Lipid diagnosis (%)	24,262	29.0	40.3	9.5 × 10^−37^
CVD diagnosis (%)	24,262	7.4	33.3	< 1.0 × 10^−300^
Cancer diagnosis (%)	24,262	6.5	19.4	3.3 × 10^−130^
Lung diagnosis (%)	24,262	11.0	16.5	4.8 × 10^−19^

Data are presented as mean ± SD, median (IQR), numbers or percentages

^1^In *n* = 19,492

^2^In *n* = 13,681

Continuous variables are presented as mean ± standard deviation, median, and interquartile range (IQR), and categorical variables are presented as percentages. A *p* value < 0.05 was used as a cutoff for statistical significance. Analyses were conducted using IBM SPSS Statistics (Version 24, IBM, Armonk, NY, USA) and Stata Statistical Software (version 16.0; Stata Corp, College Station, TX, USA).

## Results

### Baseline characteristics

The final study population had a mean age of 48 (SD 19) years; 50.1% were males. The median follow-up duration was 109 months (range 1–201 months). At the census day of December 31, 2015, 3023 participants were determined as deceased (12.5%). Reported causes of death are summarized in Additional file 3: Table 2. Participants who had died were older, more frequently males, had higher blood pressure, and more frequently abnormal laboratory values, with higher mean concentrations of serum creatinine (and lower eGFR), C-reactive protein, and homocysteine, indicating a higher degree of low-grade inflammation (Table 1). In addition, they were more frequently former smokers, and had a higher prevalence of diabetes, hypertension, CVD, cancer, or lung disease. There was no difference in serum B12 concentrations between participants who died and those who remained alive. Table 2 shows the relevant baseline variables according to serum B12 concentration. As expected, lower serum B12 coincided with higher serum MMA and homocysteine concentrations. In contrast, we observed higher serum folate concentrations in the participants with the highest serum B12 concentrations. For prevalent diagnosis of diabetes, hypertension, CVD, cancer, and lung disease, there was a significant U-shaped relationship, with the highest disease prevalence in the participants with serum B12 < 140 and > 700 pmol/l.

**Table 2:** Relevant baseline variables according to the serum B12 group

	Serum B12 level (pmol/l)				***p*** value
	< 140	140–300	300–700	> 700	
M/F	284/310	4442/4092	6827/6639	608/1060	
% females	52.2	47.9	49.3	63.5	9.0 × 10^−25^
Age 18–39 years (%)	23.1	38.2	39.2	21.1	1.0 × 10^−71^
Age 40–59 years (%)	30.3	31.3	30.0	31.3	
Age ≥ 60 years (%)	46.6	30.5	30.8	47.6	
Body mass index (kg/m^2^)	28.8 ± 6.8	29.1 ± 7.0	28.2 ± 6.5	27.9 ± 6.3	4.7 × 10^−23^
Waist circumference (cm)	100 ± 16	99 ± 16	96 ± 16	96 ± 15	1.2 × 10^−37^
SBP (mmHg)	128 ± 20	125 ± 19	124 ± 19	127 ± 21	1.0 × 10^−12^
DBP (mmHg)	70 ± 11	71 ± 12	70 ± 12	70 ± 12	1.1 × 10^−4^
C-reactive protein (mg/dl)	0.20 (0.09–0.44)	0.21 (0.08–0.50)	0.20 (0.08–0.47)	0.24 (0.09–0.56)	0.006
Serum folate (nmol/l)	34.1 (24.2–46.0)	34.3 (24.7–46.8)	41.9 (30.0–58.0)	55.1 (37.1–77.8)	3.0 × 10^−209^
Methylmalonic acid (nmol/l)^1^	272 (172–522)	150 (112–207)	124 (98–162)	122 (94–160)	3.8 × 10^−194^
Homocysteine (μmol/l)^2^	13.0 (9.5–18.3)	8.7 (7.1–11.0)	7.7 (6.4–9.4)	7.3 (5.8–9.1)	8.8 × 10^−109^
Annual family income					0.035
< 25k (%)	43.8	39.6	37.9	38.1	
25–75k (%)	39.1	40.1	41.2	40.8	
> 75k (%)	17.1	20.3	20.9	21.1	
Never smoking (%)	51.1	47.0	51.3	56.9	8.7 × 10^−15^
Former smoking (%)	21.5	20.9	21.3	25.4	0.001
Current smoking (%)	26.4	30.0	24.9	16.7	9.3 × 10^−33^
Use of vitamin B12-containing supplements (%)	17.0	20.3	35.2	59.5	2.6 × 10^−264^
Diabetes diagnosis (%)	21.9	15.2	15.3	22.8	8.6 × 10^−18^
Hypertension diagnosis (%)	43.1	34.3	32.6	45.4	4.3 × 10^−28^
Lipid diagnosis (%)	32.8	29.9	29.7	37.0	1.3 × 10^−8^
CVD diagnosis (%)	16.5	10.4	9.9	15.0	2.0 × 10^−13^
Cancer diagnosis (%)	10.1	7.6	7.9	11.5	4.8 × 10^−7^
Lung diagnosis (%)	14.5	11.7	11.3	13.9	0.002

Data are presented as mean ± SD, median (IQR), numbers, or percentages

^1^In *n* = 19,492

^2^In *n* = 13,681

### Trends over time 1999–2014

For some variables, there was a relevant change over time with subsequent NHANES surveys. Current smoking remained rather stable between 1999 and 2000 and 2013–2014, amounting 19.5 and 20.4% of participants, respectively (*p* = 0.137), although there was a gradual decrease in the percentage of former smokers (25.9–26.9% between 1999 and 2000 and 2003–2004 vs 23.1% in 2011–2012 and 2013–2014, *p* < 0.001). The mean BMI increased from 28.3 ± 6.3 kg/m^2^ in 1999–2002 to 29.0 ± 7.0 kg/m^2^ in 2011–2014. There was a significant increase in the percentage of participants with a diagnosis of diabetes (11.8% in 1999–2000, 19.4% in 2013–2014, *p* < 0.001), hypertension (34.7% vs 39.6%, *p* < 0.0001), cancer (6.3% vs 9.2%, *p* < 0.001), and lung disease (7.5% vs 13.3%, *p* < 0.001). Also, we observed a significant increase in the use of high-dose vitamin B12-containing supplements: in the surveys of 1999–2002, 2.7–3.3% were taking supplements containing ≥ 100 mcg cobalamin and 0.1–0.2% ≥ 1000 mcg. In the surveys 2011–2012 and 2013–2014, this had increased to 5.9–6.7% and 2.9–3.1%, respectively.

### Association with mortality

**Fig. 2:**
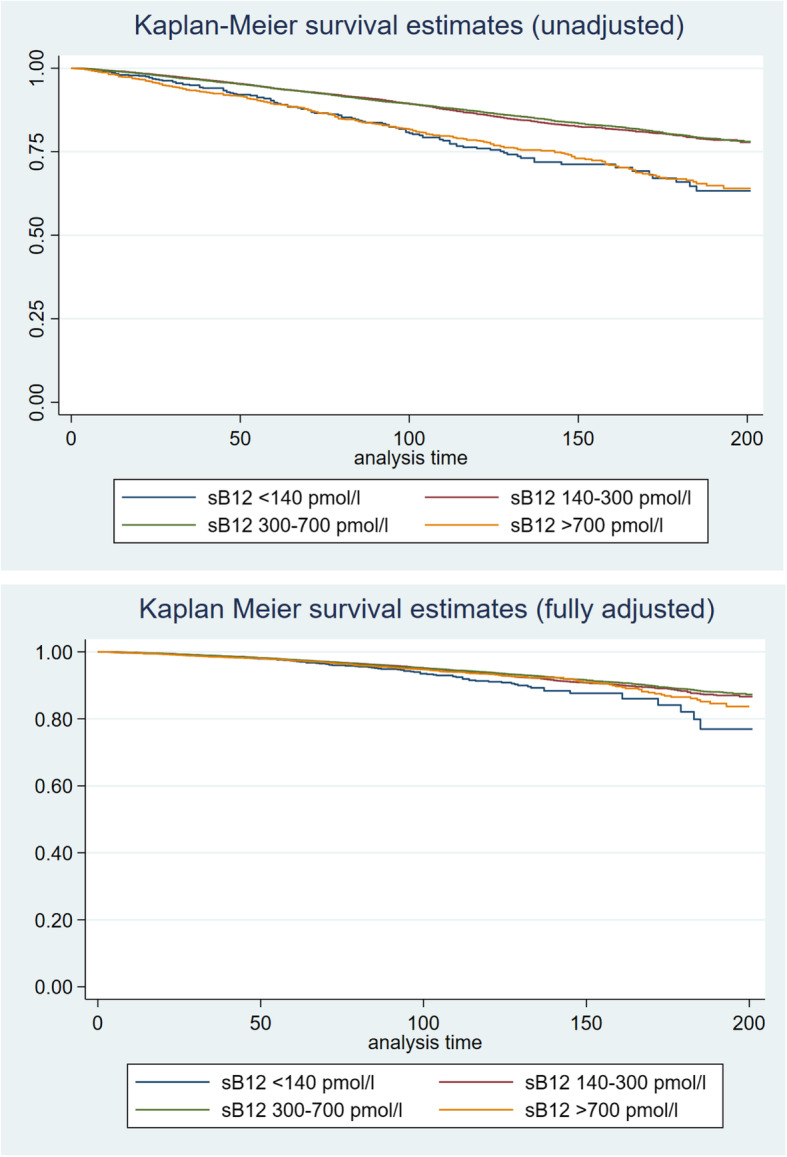
Kaplan-Meier survival estimates for the serum B12 groups, unadjusted (top) and fully adjusted for covariates (bottom)

**Fig. 3:**
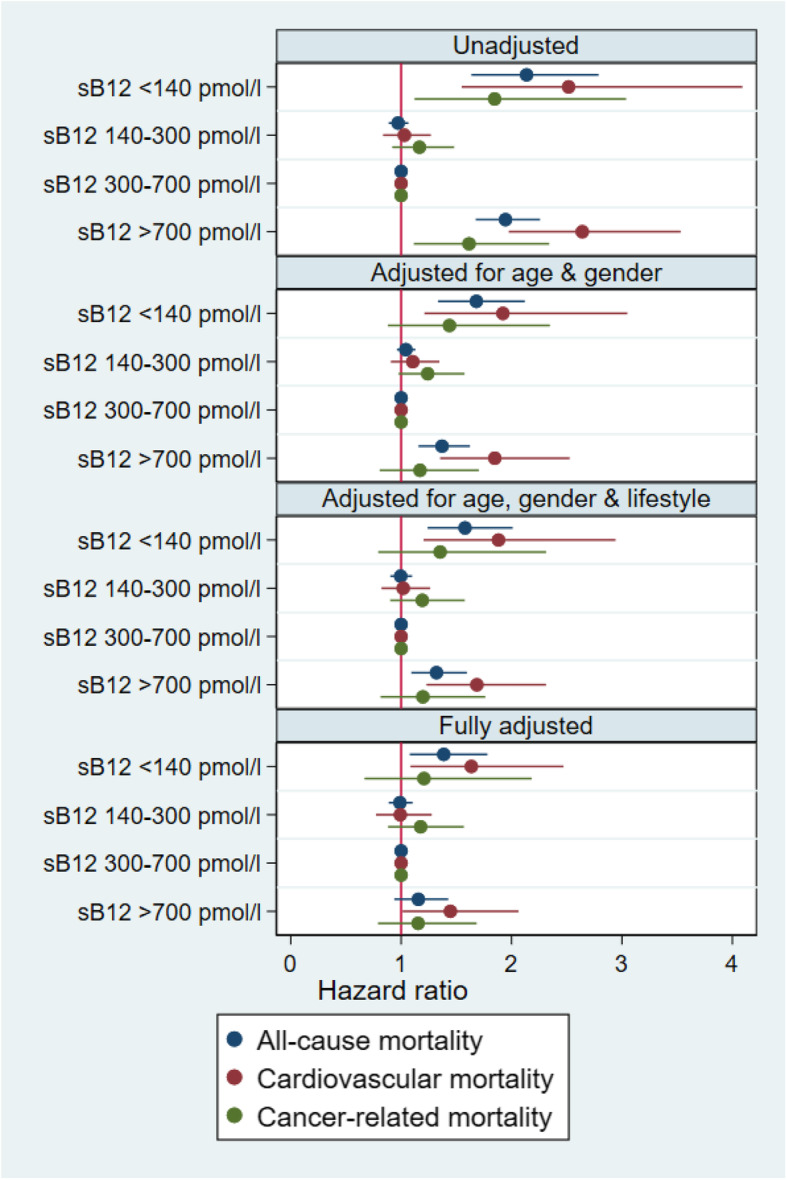
Cox proportional hazards analysis for all-cause, cardiovascular, and cancer-related mortality according to serum B12 concentrations in 19,034 participants of the NHANES surveys 1999–2014 with complete data. Data show hazard ratio and 95% CI, for 4 models. Panel 1, unadjusted. Panel 2, adjusted for age (categorical, < 40, 40–60, > 60), gender, and ethnicity. Panel 3, adjusted for age, gender, ethnicity, BMI group (categorical, < 20, 20–25, 25–30, 30–35, 35–40, > 40), family income (categorical, < $25,000, $25000–75,000, > $75,000), education level, former and current smoking, and alcohol consumption. Panel 4, adjusted for age; gender; ethnicity; BMI group; family income; education level; former and current smoking; alcohol consumption; eGFR < 60 ml/min/1.73 m^2^; diagnosis of diabetes, hypertension, cardiovascular disease, cancer, and lung disease; medication use (as a proxy for other comorbidities); white blood cell count; hemoglobin; and serum folate

The association of serum B12 concentrations with mortality was U-shaped, with significantly higher mortality with low as well as with high serum B12 (Fig. 1, Additional file 4: Table 3). The Kaplan-Meier analysis revealed statistically significant (*p* < 0.001, Fig. 2, top) differences in survival probabilities across the four serum B12 groups. The unadjusted analysis indicated the lowest survival in participants with serum B12 < 140 pmol/l and > 700 pmol/l. After adjustment for all relevant confounders (Fig. 2, bottom), only the survival curve for participants with serum B12 < 140 pmol/l deviated from the 3 other curves. These observations are confirmed by the detailed Cox proportional hazard analyses (Fig. 3). In unadjusted analyses, both low and high serum B12 concentrations were associated with a higher odds ratio of all-cause and cardiovascular mortality. The adjusted analyses indicated that low serum B12 < 140 pmol/l remained significantly associated with all-cause and cardiovascular mortality, while high serum B12 > 700 pmol/l was associated with increased cardiovascular mortality only. In none of the adjusted models could we demonstrate a relationship between serum B12 concentrations and cancer-related mortality.

**Table 3:** Distribution of the use of oral vitamin B12-containing supplements and concomitant serum B12 concentrations

Vitamin B12 supplements	Number	Serum B12 (pmol/l)			Serum B12 < 140	Serum B12 > 1000
		Median (IQR)	Minimum	Maximum		
No supplements	16,385	321 (242–423)	10.5	148,936	488 (3.0%)	211 (1.3%)
0–4.9 mcg	1115	345 (258–458)	75.3	9810	22 (2.0%)	19 (1.7%)
5–24.9 mcg	3358	391 (294–523)	25.1	75,645	47 (1.4%)	83 (2.5%)
25–99 mcg	1774	428 (321–564)	36.9	4794	24 (1.4)	61 (3.4%)
100–999 mcg	780	502 (373–696)	86.4	7557	4 (0.5%)	80 (10.3%)
> 1000 mcg	387	732 (495–1198)	103	8576	3 (0.8%)	125 (32.3%)
Unknown	463	377 (280–548)	34.7	33,837	6 (1.3%)	26 (5.6%)

Participants reporting the use of B12 injections under dietary supplements or prescription medications were excluded

### Vitamin B12-containing supplements

**Fig. 4:**
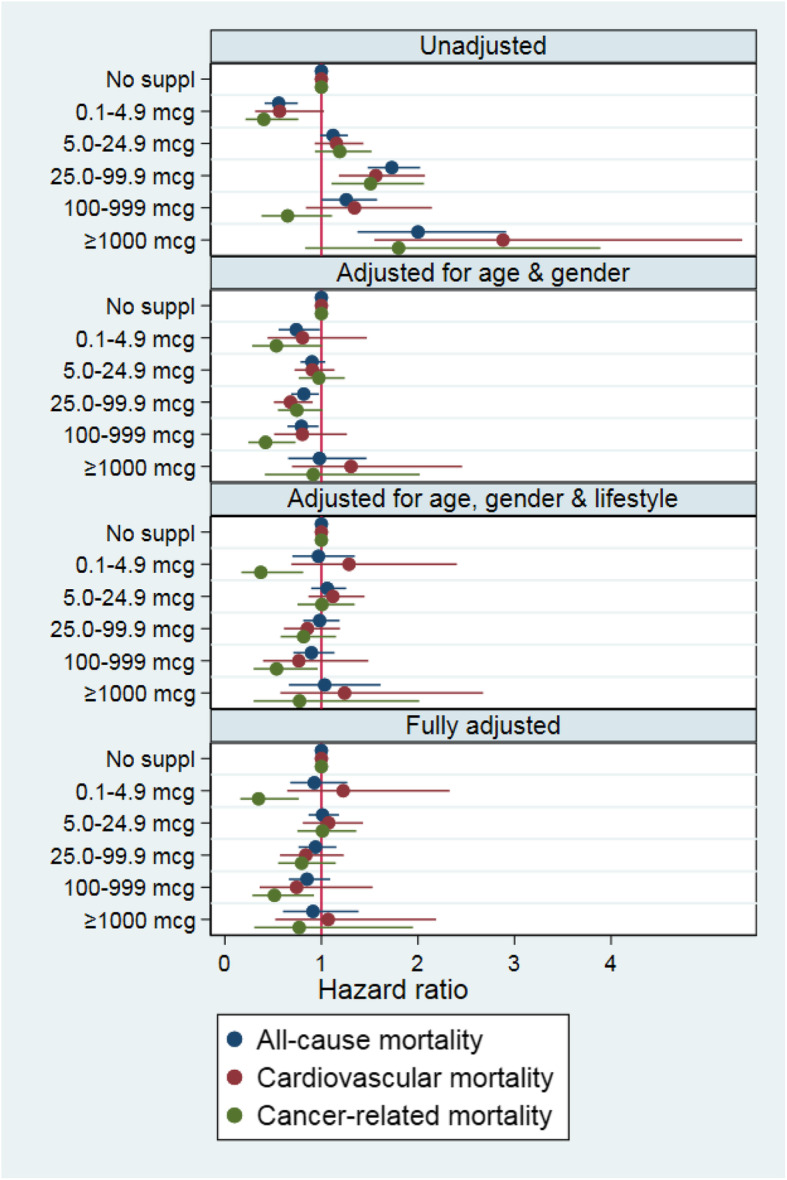
Association between the use of oral vitamin B12 supplement intake (in mcg per day) and hazard ratio for all-cause, cardiovascular, and cancer-related mortality in 18,666 participants of the NHANES surveys 1999–2014 with complete data. Panel 1, unadjusted. Panel 2, adjusted for age (categorical, < 40, 40–60, > 60), gender, and ethnicity. Panel 3, adjusted for age, gender, ethnicity, BMI group (categorical, < 20, 20–25, 25–30, 30–35, 35–40, > 40), family income (categorical, < $25,000, $25,000–75,000, > $75,000), education level, former and current smoking, and alcohol consumption. Panel 4, adjusted for age; gender; ethnicity; BMI group; family income; education level; former and current smoking; alcohol consumption; eGFR < 60 ml/min/1.73 m^2^; diagnosis of diabetes, hypertension, cardiovascular disease, cancer, and lung disease; medication use (as a proxy for other comorbidities); white blood cell count; hemoglobin; and serum folate

In total, 7877 participants reported the use of oral vitamin B12-containing supplements in any form. Users of these supplements more frequently had a diagnosis of hypertension (35.7 vs 28.8%), hyperlipidemia (35.6 vs 29.2%), CVD (34.1 vs 30.8%), and cancer (44.8 vs 29.9%, all *p* < 0.001). A higher intake of vitamin B12 by supplements coincides with higher serum B12 concentrations (Table 3). Nevertheless, all groups contain participants with markedly elevated serum B12 concentrations. Although the unadjusted Cox proportional hazards analysis suggested a difference in all-cause mortality between different supplemental B12 intakes (Fig. 4), none of the adjusted models showed increased mortality in relation to intake of vitamin B12-containing supplements.

### Sensitivity analyses

The hazard ratio of low serum B12 for both all-cause and cardiovascular mortality in the fully adjusted model is similar in the 1999–2006 participants compared to the entire cohort (Additional file 5: Table 4). However, it is no longer significant when adjusted additionally for the powerful biomarkers homocysteine and MMA which increase when there is symptomatic or functional vitamin B12 deficiency. The association between high serum B12 and all-cause mortality has a comparable hazard ratio for all sensitivity analyses.

## Discussion

Our unadjusted data showed a U-shaped association between serum B12 concentrations and subsequent all-cause mortality after a median follow-up duration of 109 months in NHANES participants aged 18 and above. After adjusting for relevant confounders, including socioeconomic status, education, ethnicity, smoking, comorbidity, and laboratory parameters, low serum B12 was associated with a moderate increase in all-cause and cardiovascular mortality. Sensitivity analyses revealed that this effect was no longer significant when adjusted for the relevant biomarkers homocysteine and MMA, which indicate a tissue B12 deficiency. High serum B12 concentrations > 700 pmol/l were associated with an increase in cardiovascular mortality only (HR 1.45, 95% CI 1.01–2.06, *p* = 0.042). Participants with a diagnosis of hypertension, dyslipidemia, CVD, and cancer more frequently used vitamin B12-containing supplements than those without these diagnoses. Congruently, we found no indication that a higher intake of vitamin B12-containing supplements was associated with adverse effects on mortality.

Traditionally, it was believed that the principal and inevitable consequence of vitamin B12 deficiency was megaloblastic anemia as part of a well-characterized syndrome that was described decades ago. Current evidence, however, suggests that frequently hematological manifestations are entirely absent [1, 4]. There is only limited information on the long-term consequences of low serum B12 concentrations. A study in Finland showed an association between the lowest tertile of serum B12 concentrations and the risk of mortality due to vascular events or dementia [30]. A Dutch study could not confirm this, but these observations comprised only a small group of patients after myocardial infarction, and serum B12 concentrations were measured soon after the event [31]. A review by Rafnsson concluded that there was limited evidence that vitamin B12 deficiency predisposes to the risk of mortality and morbidity from either cardiovascular diseases or diabetes in adults [32]. Data from the LURIC study, however, showed a similar U-shaped curve for the association between serum B12 and mortality as in the present study [33]. These authors proposed that in low serum B12, mortality—and the accompanying accelerated telomere shortening—might be driven by increased concentrations of homocysteine, while in participants with high serum B12, this might be related to increased inflammation. Since the advent of mandatory fortification of the food supply with folic acid in the USA in 1998, vitamin B12 deficiency has become the leading modifiable cause of elevated homocysteine levels in the population [34]. These results are confirmed by our sensitivity analyses, in which the hazard ratio of low serum B12 for both all-cause and cardiovascular mortality is no longer significant when adjusted additionally for homocysteine concentrations.

The (epidemiologic) relationship of increased serum B12 concentrations and subsequent disease or mortality has been the topic of several papers (Additional file 6: Table 5), showing the putative prognostic significance of increased serum B12 concentrations in people who were acutely ill. Several studies reported that high serum B12 concentrations predicted mortality in alcoholic hepatitis [35], inpatients [36–40], and in those critically ill [41]. In contrast, in patients with ischemic stroke, low serum B12 was associated with lower Glasgow Coma Scale scores and higher mortality [42]. Elevated serum B12 was associated with severity of disease in acute-on-chronic liver failure [43], interstitial renal disease, liver cirrhosis, hepatitis, and liver metastases [44] and proved to be a marker of poor liver function, but not a predictor of mortality after admission to an intensive care unit [45]. These data altogether suggest that serum B12 concentrations increase as a consequence of acute and chronic illness. Several hypotheses have been postulated regarding the mechanism behind this, including an upregulation of haptocorrin synthesis, an increased release of cellular cobalamin, for instance in liver disorders during hepatic cytolysis, or a reduction of cobalamin clearance by a diseased liver [46]. The associations that have been reported by others between high serum B12 concentrations and morbidity or mortality and the proposed underlying mechanism of such associations are plausible, invoking, as they do, a primary disease process that leads to secondary elevation of circulating B12 concentrations, presumably through tissue release of vitamin B12. However, there is nothing in those reports that indicates causality in the direction of a priori raised serum B12 leading to increased mortality. While the findings in our study confirm the overall U-shaped association of serum B12 concentration with mortality, they lend no support to the suggestion that high serum B12 concentrations per se are harmful or detrimental.

Studies in people who were not acutely ill showed varying results. Most authors reported no association between serum B12 and cardiovascular disease or death due to coronary heart disease [8–12], one study reported inconsistent results [47], while some reported increased all-cause mortality with higher serum B12 [13, 48, 49]. The reports in cancer are even more confusing. In one study, higher serum B12 indicated increased prostate cancer risk [50], but this was not confirmed in another study [51]. Higher serum B12 predicted lower overall survival in patients with metastatic cancer [52]. Plasma folate and B12 concentrations were not associated with breast cancer risk in women recruited to the Varese (Italy) cohort of the European Prospective Investigation into Cancer and Nutrition (EPIC) study, while high plasma vitamin B6 was associated with lower risk [53]. Serum B12 concentrations were not associated with lung cancer risk in a large meta-analysis [54]. In contrast, a recent paper suggested that increasing serum B12 concentrations were associated with an increased risk for lung cancer [7]. A doubling of B12 concentrations was calculated to be associated with a 15% increase of risk. The increased risk was not observed in participants who never smoked. Interestingly, the reported increased risk for lung adenocarcinoma was associated with the specific effect of specific single nucleotide polymorphisms (SNPs), which increase circulating serum B12 [7]. One of these is a SNP in the gene fucosyltransferase 2 (FUT2), where the FUT2 non-secretor rs601338(AA) variant is associated with a 10–25% higher total and haptocorrin-bound B12, but not transcobalamin-bound B12 [55].

Our current paper could not demonstrate any relationship between dietary vitamin B12 intake and cancer mortality. In previous reports, dietary intake of vitamin B12 (as well as folate and B6) or B12 supplement use was not associated with increased risk of renal [56], nasopharyngeal [57], or lung cancer [14] and was associated with a lower risk of pancreatic cancer [58] and decreased risk of breast cancer in BRCA mutation carriers [59]. In the Women’s Antioxidant and Folic Acid Cardiovascular Study, intervention with a combination of folic acid (2.5 mg daily), vitamin B6 (50 mg), and cyanocobalamin (1000 mcg) for 7.3 years had no effect on the overall risk of cancer or cancer mortality [60]. In contrast, the combined analysis of two studies in Norway—where there is no folic acid fortification of foods—showed that treatment of patients with ischemic heart disease using folic acid (0.8 mg) plus cyanocobalamin (400 mcg) was associated with increased cancer risk and all-cause mortality. The authors suggest that these adverse effects were mediated by folic acid [61]. Supplementation with folate, vitamins B6 and B12, and/or omega-3 fatty acids in a secondary prevention trial showed no benefit of low doses of vitamin B6 (3 mg), cyanocobalamin (20 mcg), and 5-methyltetrahydrofolate (0.56 mg) on cancer incidence or cancer mortality in patients with cardiovascular disease [62].

An important question, which cannot be answered with the current dataset, is whether and why many NHANES participants might have taken extra vitamin B12-containing supplements. Several participants took high doses of vitamin B12, in the range of 100 to 6000 mcg per day, while the recommended daily allowance (RDA) for adults is 2.4 mcg/day. For some, this might have been a treatment of demonstrated vitamin B12 deficiency or additional nutrient supplementation in the case of vegetarians, for others, such supplement use was either self-driven or by the recommendation of a health care provider. We clearly showed that the use of vitamin B12-containing supplements has increased during the more recent NHANES surveys and was significantly higher in participants with hypertension, dyslipidemia, CVD, and an earlier diagnosis of cancer. The association between elevated serum B12 and cardiovascular mortality, together with the absence of any association between high B12 supplement intake and mortality likely indicates “reverse causality.” The increased use of vitamin B12-containing supplements by people with a chronic (cardiovascular) condition supports this conclusion, as well as the growing body of literature that serum B12 concentrations increase in states of inflammation [33]. However, there are several other factors which by themselves may negatively influence long-term morbidity and mortality, and are associated with higher serum B12 concentrations, for instance, high consumption of red meat. A higher intake of processed meat and unprocessed red meat may be associated with a small increased risk of incident CVD and all-cause mortality [63–65]. In the dataset, we observed several individuals with markedly elevated serum B12 concentrations which were not explained by oral supplement use or registered cobalamin-containing prescription medication. This could suggest “hidden” or non-reported use of such preparations or injectable use of vitamin B12 (personal communication prof. R.K. Bailey), but it may also indicate other factors which may lead to artificially elevated serum B12 concentrations such as recent infection, or the existence of circulating B12 immune complexes [66, 67].

Our sensitivity analyses confirm the robustness of our findings. The hazard ratio of low serum B12 for both all-cause and cardiovascular mortality is similar in a subset of participants, i.e., those included between 1999 and 2006, but is no longer significant when adjusted additionally for homocysteine or MMA concentrations. Both have been considered biomarkers with high sensitivity, and in the case of MMA, also specificity, indicating B12 deficiency at the tissue level [68], although several authors have reported normal MMA values even with severely low serum B12 concentrations [5, 69–71]. Previously, we have shown in NHANES 2011–2014 participants that serum MMA concentrations were a more reliable predictor of complaints, functional status, and physical performance than serum B12 [16]. This may also explain why the association between serum B12 and mortality loses its significance when adjusted for MMA concentrations. The observed association between high serum B12 and cardiovascular mortality has a comparable hazard ratio of 1.41–1.46 for all sensitivity analyses but lost its significance due to a lower number of participants with complete data evaluated in the 1999–2006 surveys (Additional file 5: Table 4).

Prospective follow-up studies do have pitfalls. Dietary habits recorded at baseline may significantly change during the course of several years of follow-up, especially when people develop intercurrent disorders like myocardial infarction or diabetes. Also, socioeconomic situations may improve or worsen, and alterations in exercise and other environmental factors like sleeping habits or sleep quality or medication use may affect the development of morbidity and death. In the current analysis in NHANES, no follow-up data other than mortality were available. Future studies in which repeated measurements and questionnaires are taken over time may validate the reported associations. Such studies are however very expensive to organize and perform. Despite this, the current analyses have tried to incorporate as far as possible the relevant confounders for the development of morbidity and mortality, including socioeconomic status, education, ethnicity, comorbidity, and laboratory parameters, thereby showing the strength of this very large NHANES dataset.

## Conclusions

After adjustment for relevant demographic and lifestyle factors as well as existing comorbidity, low serum B12 concentrations were independently associated with a slight increase of all-cause and cardiovascular mortality. Elevated serum B12 was associated with a small increase in cardiovascular mortality only. High intake of vitamin B12 in the form of supplements was not associated with increased all-cause mortality, supporting its safe use. Vitamin B12 deficiency is prevalent, particularly among the elderly, and is associated with serious morbidity. The diagnosis of such deficiency is often elusive so that the use of B12 supplements has become widespread, because of its low cost and presumed safety. Recent reports based on associations have raised the specter that high B12 levels may be harmful and have discouraged B12 supplement use with potentially harmful consequences. The overall public health impact of our findings is to provide reassurance that high B12 levels per se are not a cause of higher mortality risk.

## Supplementary information

**Additional file 1: Figure S1.** Flow chart of the study population. Describes how the present sample of participants was composed.

**Additional file 2: Table S1.** Statistical analysis: confounders used in the Cox proportional hazards analysis. Describes the important confounders which have been used in the adjusted cox proportional hazard models, and the use of the specific sampling weights that NHANES has created to account for its complex survey design (including oversampling), survey non-response, and post-stratification.

**Additional file 3: Table S2.** Underlying cause of death according to NCHS definitions. Summarizes the underlying causes of death as registered in the NHANES linked National Death Index public-access files through December 31, 2015.

**Additional file 4: Table S3.** Serum B12 concentrations and mortality**.** Describes the percentage of participants who died due to cardiovascular, cancer and other causes stratified for serum b12 concentration < 140, 140–300, 300–700, and > 700 pmol/l.

**Additional file 5: Table S4.** Sensitivity analysis for Cox proportional hazard analysis for groups of serum B12 concentrations**.** This file describes the sensitivity analyses, in which we recalculated the Cox PH model for all-cause mortality in only participants of the 1999–2006 surveys (without and with inclusion of homocysteine concentrations and C-reactive protein as additional adjustment for chronic inflammation), and separately only for those participants in whom also serum concentrations of MMA were available.

**Additional file 6: Table S5.** Summary of the most important literature on the association of serum B12 concentrations and disease or mortality

## Data Availability

The datasets generated and analyzed during the current study are available on the NHANES website: https://www.cdc.gov/nchs/nhanes/index.htm

## Abbreviations

BMI: Body mass index
CHD: Coronary heart disease
CVD: Cardiovascular disease
DBP: Diastolic blood pressure
eGFR: Estimated glomerular filtration rate
EPIC: European Prospective Investigation into Cancer and Nutrition
FUT2: Fucosyltransferase 2
HbA1c: Glycated hemoglobin
HCys: Homocysteine
HR: Hazard ratio
ICU: Intensive care unit
MMA: Methylmalonic acid
NHANES: National Health and Nutrition Examination Survey
PH: Proportional hazards
SBP: Systolic blood pressure
SNP: Single nucleotide polymorphism
WBC: White blood cells
WHI: Women’s Health Initiative
