# Supplementary material for: Relationship between serum B12 concentrations and mortality: experience in NHANES
Source: BMC Med. 2020 Oct 9;18:307. doi: 10.1186/s12916-020-01771-y (PMC7545540; doi:10.1186/s12916-020-01771-y)
Supplement: Supplementary file 1 — Additional file 1: Figure S1. Flow chart of the study population. Describes how the present sample of participants was composed. [file 12916_2020_1771_MOESM1_ESM.docx]

Additional file 1: Figure 1. Flow chart of the study population.

**NHANES 1999-2014**

n = 82901 participants

Eligible for mortality follow-up:

n = 47279

Availability of serum B12 measurement, and not reporting being pregnant:

n = 24262

Full dataset for Cox PH model according to serum B12 concentrations:

n = 19034

Full dataset for Cox PH model according to use of vitB12 supplements:

n = 18666
