# Supplementary material for: Relationship between serum B12 concentrations and mortality: experience in NHANES
Source: BMC Med. 2020 Oct 9;18:307. doi: 10.1186/s12916-020-01771-y (PMC7545540; doi:10.1186/s12916-020-01771-y)
Supplement: Supplementary file 2 — Additional file 2: Table S1. Statistical analysis: confounders used in the Cox proportional hazards analysis. Describes the important confounders which have been used in the adjusted cox proportional hazard models, and the use of the specific sampling weights that NHANES has created to account for its complex survey design (including oversampling), survey non-response, and post-stratification. [file 12916_2020_1771_MOESM2_ESM.docx]

Additional File 2: Table 1. Statistical analysis: confounders used in the Cox proportional hazards analysis.

**Demographic factors:**

age (18-39 years, 40-59 years, ≥60 years); gender; ethnicity (Non-Hispanic White, Non-Hispanic Black, Mexican American, Other Hispanic, Other Race);

**Socioeconomic and lifestyle factors:**

body mass index (BMI; <20, 20 to <25, 25 to <30, 30 to <35, 35 to <40, ≥40 kg/m2); education (less than 9th grade, 9–11th grade, high-school grade, some college or associate's (AA) degree, college graduate and above); annual family income (<$25,000, $25,000 to $75,000, >$75,000); smoking (non-smoker, former smoker, current smoker); alcohol consumption;

**Comorbidities and laboratory measurements:**

a. diagnosis of diabetes mellitus (defined as self-reported diabetes, hemoglobin A1c ≥ 6.5%, fasting plasma glucose level ≥7.0 mmol/l, or the use of oral glucose-lowering medication or insulin);

b. hypertension (self-reported hypertension, systolic blood pressure ≥140 mm Hg, or diastolic blood pressure ≥90 mm Hg or reported use of medication to lower blood pressure);

c. dyslipidemia (total cholesterol ≥ 6.0 mmol/l, low-density cholesterol ≥ 3.5 mmol/l, or use of statins);

d. chronic kidney disease (glomerular filtration rate <60 mL/min/1. 73 m^2^);

e. previous cardiovascular disease (self-reported ischemic heart disease, heart failure or previous stroke);

f. self-reported history of cancer;

g. self-reported pulmonary disorders (asthma, emphysema, chronic bronchitis);

h. other comorbidities, based on chronic medication use;

i. white blood cell count and hemoglobin, as a proxy for inflammation;

j. serum folate concentrations.

NHANES has created specific sampling weights to account for its complex survey design (including oversampling), survey non-response, and post-stratification [1]. The incorporation of sampling weights into estimated regression coefficients helps to protect against the potential existence of missing regressors. In addition, the linearization variance estimator is suggested to be robust against the likelihood of correlated errors and the possibility of heteroscedasticity [2,3]. Although we did not intend to extrapolate our findings to the U.S. civilian noninstitutionalized Census population, we calculated our multivariable regression models with application of these weights.

References:

1. NHANES tutorials. https://wwwn.cdc.gov/nchs/nhanes/tutorials/default.aspx. Accessed June 10.

2. Kott PS: A Model-Based Look at Linear Regression with Survey Data. The American Statistician 1991, 45(2):107-112.

3. Gallagher CM, Smith DM, Meliker JR: Total blood mercury and serum measles antibodies in US children, NHANES 2003-2004. Sci Total Environ 2011, 410-411:65-71.
