# Supplementary material for: Relationship between serum B12 concentrations and mortality: experience in NHANES
Source: BMC Med. 2020 Oct 9;18:307. doi: 10.1186/s12916-020-01771-y (PMC7545540; doi:10.1186/s12916-020-01771-y)
Supplement: Supplementary file 3 — Additional file 3: Table S2. Underlying cause of death according to NCHS definitions. Summarizes the underlying causes of death as registered in the NHANES linked National Death Index public-access files through December 31, 2015. [file 12916_2020_1771_MOESM3_ESM.docx]

Additional File 3: Table 2. Underlying cause of death according to NCHS definitions

Underlying Cause of Death: Recode

Diseases of heart (I00-I09, I11, I13, I20-I51) 570

Malignant neoplasms (C00-C97) 629

Chronic lower respiratory diseases (J40-J47) 144

Accidents (unintentional injuries) (V01-X59, Y85-Y86) 114

Cerebrovascular diseases (I60-I69) 133

Alzheimer's disease (G30) 85

Diabetes mellitus (E10-E14) 98

Influenza and pneumonia (J09-J18) 62

Nephritis, nephrotic syndrome and nephrosis

(N00-N07, N17-N19, N25-N27) 54

All other causes (residual) 1126

Unknown 8

Total 3023
