# Supplementary material for: Relationship between serum B12 concentrations and mortality: experience in NHANES
Source: BMC Med. 2020 Oct 9;18:307. doi: 10.1186/s12916-020-01771-y (PMC7545540; doi:10.1186/s12916-020-01771-y)
Supplement: Supplementary file 4 — Additional file 4: Table S3. Serum B12 concentrations and mortality. Describes the percentage of participants who died due to cardiovascular, cancer and other causes stratified for serum b12 concentration < 140, 140–300, 300–700, and > 700 pmol/l. [file 12916_2020_1771_MOESM4_ESM.docx]

Additional File 4: Table 3. Serum B12 concentrations and mortality

|  | **Mortality** | | | |
| --- | --- | --- | --- | --- |
| **Serum B12 concentrations** | **All-cause** | **Cardiovascular** | **Cancer** | **Other causes** |
| < 140 pmol/l | 18.2 | 4.2 | 4.0 | 10.0 |
| 140-300 pmol/l | 12.0 | 2.9 | 2.8 | 6.3 |
| 300-700 pmol/l | 11.8 | 2.7 | 2.3 | 6.8 |
| > 700 pmol/l | 17.5 | 4.8 | 3.5 | 9.2 |
