# Supplementary material for: Relationship between serum B12 concentrations and mortality: experience in NHANES
Source: BMC Med. 2020 Oct 9;18:307. doi: 10.1186/s12916-020-01771-y (PMC7545540; doi:10.1186/s12916-020-01771-y)
Supplement: Supplementary file 5 — Additional file 5: Table S4. Sensitivity analysis for Cox proportional hazard analysis for groups of serum B12 concentrations. This file describes the sensitivity analyses, in which we recalculated the Cox PH model for all-cause mortality in only participants of the 1999–2006 surveys (without and with inclusion of homocysteine concentrations and C-reactive protein as additional adjustment for chronic inflammation), and separately only for those participants in whom also serum concentrations of MMA were available. [file 12916_2020_1771_MOESM5_ESM.docx]

Additional File 5: Table 4. Sensitivity analysis for Cox proportional hazard analysis for groups of serum B12 concentrations

| **Serum B12 concentrations** | **Full cohort**  **analysis ^a^** | **Surveys 1999-2006 ^b^** | **Surveys 1999-2006 + HCYS/CRP ^c^** | **Full cohort with MMA ^d^** |
| --- | --- | --- | --- | --- |
| Complete data | n=19034 | n=10738 | n=10711 | n=15324 |
| < 140 pmol/l | 1.39 (1.08-1.78)  p=0.011 | 1.42 (1.09-1.85)  p=0.010 | 1.09 (0.82-1.46)  p=0.556 | 1.18 (0.87-1.58)  p=0.284 |
| 140-300 pmol/l | 0.99 (0.89-1.10)  p=0.850 | 0.99 (0.88-1.12)  p=0.929 | 0.96 (0.85-1.08)  p=0.500 | 0.97 (0.85-1.09)  p=0.589 |
| 300-700 pmol/l | Reference | Reference | Reference | Reference |
| > 700 pmol/l | 1.16 (0.94-1.43)  p=0.172 | 1.13 (0.89-1.42)  p=0.311 | 1.13 (0.89-1.44)  p=0.302 | 1.14 (0.91-1.44)  p=0.248 |
|  |  |  |  |  |
| **Serum B12 concentrations** | **Full cohort**  **analysis ^a^** | **Surveys 1999-2006 ^b^** | **Surveys 1999-2006 + HCYS/CRP ^c^** | **Full cohort with MMA ^d^** |
| < 140 pmol/l | 1.64 (1.08-2.47)  p=0.020 | 1.62 (1.08-2.45)  p=0.022 | 1.16 (0.70-1.94)  p=0.558 | 1.39 (0.80-2.40)  p=0.235 |
| 140-300 pmol/l | 0.99 (0.77-1.28)  p=0.954 | 1.00 (0.77-1.31)  p=0.998 | 0.95 (0.73-1.24)  p=0.703 | 0.98 (0.75-1.28)  p=0.883 |
| 300-700 pmol/l | Reference | Reference | Reference | Reference |
| > 700 pmol/l | 1.45 (1.01-2.06)  p=0.042 | 1.46 (1.00-2.14)  p=0.052 | 1.46 (0.98-2.18)  p=0.063 | 1.41 (0.94-2.13)  p=0.093 |
|  |  |  |  |  |
| **Serum B12 concentrations** | **Full cohort**  **analysis ^a^** | **Surveys 1999-2006 ^b^** | **Surveys 1999-2006 + HCYS/CRP ^c^** | **Full cohort with MMA ^d^** |
| < 140 pmol/l | 1.21 (0.67-2.18)  p=0.529 | 1.27 (0.66-2.42)  p=0.469 | 1.07 (0.52-2.20)  p=0.847 | 1.65 (0.93-2.93)  p=0.085 |
| 140-300 pmol/l | 1.18 (0.88-1.57)  p=0.264 | 1.14 (0.84-1.56)  p=0.391 | 1.12 (0.81-1.53)  p=0.484 | 1.38 (1.01-1.87)  p=0.043 |
| 300-700 pmol/l | Reference | Reference | Reference | Reference |
| > 700 pmol/l | 1.15 (0.79-1.68)  p=0.455 | 1.01 (0.68-1.51)  p=0.948 | 1.02 (0.69-1.50)  p=0.940 | 1.28 (0.83-1.98)  p=0.258 |

Data show hazards ratio and 95% CI

a: adjusted for age, gender, ethnicity, BMI group, family income, education level, former and current smoking alcohol consumption, eGFR < 60 ml/min/1.73m2, diagnosis of diabetes, hypertension, cardiovascular disease, cancer, lung disease, medication use (as a proxy for other comorbidity), white blood cell count, hemoglobin, serum folate

b: adjusted for age, gender, ethnicity, BMI group, family income, education level, former and current smoking alcohol consumption, eGFR < 60 ml/min/1.73m2, diagnosis of diabetes, hypertension, cardiovascular disease, cancer, lung disease, medication use (as a proxy for other comorbidity), white blood cell count, hemoglobin, serum folate (1999-2006 surveys only)

c: adjusted for age, gender, ethnicity, BMI group, family income, education level, former and current smoking alcohol consumption, eGFR < 60 ml/min/1.73m2, diagnosis of diabetes, hypertension, cardiovascular disease, cancer, lung disease, medication use (as a proxy for other comorbidity), white blood cell count, hemoglobin, serum folate, as well as homocysteine and serum C-reactive protein (the latter being available in n=13681 participants from 1999-2006 surveys only)

d: adjusted for age, gender, ethnicity, BMI group, family income, education level, former and current smoking alcohol consumption, eGFR < 60 ml/min/1.73m2, diagnosis of diabetes, hypertension, cardiovascular disease, cancer, lung disease, medication use (as a proxy for other comorbidity), white blood cell count, hemoglobin, serum folate, serum MMA (the latter being available in n=19492 participants from surveys 1999-2004, 2011-2014)
