# Supplementary material for: Relationship between serum B12 concentrations and mortality: experience in NHANES
Source: BMC Med. 2020 Oct 9;18:307. doi: 10.1186/s12916-020-01771-y (PMC7545540; doi:10.1186/s12916-020-01771-y)
Supplement: Supplementary file 6 — Additional file 6: Table S5. Summary of the most important literature on the association of serum B12 concentrations and disease or mortality [file 12916_2020_1771_MOESM6_ESM.docx]

Additional File 6: Table 5. Summary of the most important literature on the association of serum B12 concentrations and disease or mortality

| I**llness or hospitalization** | **Patient population** | **Average age (yrs)** | **Results** | **Country of study** |
| --- | --- | --- | --- | --- |
| Baker 1987 [1] | 370 pat’s with alcoholic hepatitis | N.A. | Increased serum B12 in alcoholic hepatitis associated with higher disease severity and mortality | USA |
| Salles 2005, 2008 [2, 3] | 488 consecutive geriatric admissions | 84.7 | Higher B12 > 400 pmol/l predicted mortality adjusted for sex and presence of cancer (both publications same cohort of patients) | France |
| Tal 2010 [4] | 1570 pat’s > 65 yrs | 81.6 | Higher serum B12 >350 pmol/l at hospitalization associated with greater in-hospital mortality | Israel |
| Sviri 2012 [5] | 663 pat’s critically ill and admitted to ICU | 60.4 | High serum B12 associated with increased mortality, 1719 pg/ml in non-survivors vs 1003 pg/ml in survivors; B12 concentrations positively correlated with severity of illness (APACHE II score). | Israel |
| Bayir 2010 [6] | 87 pat’s with ischemic stroke | 65 | Low serum B12 associated with higher mortality | Turkey |
| Dou 2012 [7] | 105 pat’s acute-on-chronic liver failure, 44 controls | 47.2 (pat’s); 45.3 (controls) | Elevated B12 concentrations associated with increased severity of liver disease and 3-month mortality rate | China |
| Jammal 2013 [8] | 3702 hospital admissions | 74.2 | High serum B12 associated with tumors, malignant hematologic diseases, metastasis, liver metastasis, liver carcinoma (LC), liver tumors other than LC, and lymphoma; low B12 associated with myeloma | France |
| Callaghan 2014 [9] | 1684 ICU admissions | 67.7 | No significant association between serum B12 and mortality after adjustment for liver function and liver disease | USA |
| Van der Wal 2015 [10] | 610 pat’s with chronic heart failure | 68 | In multivariable proportional hazard models, serum B12 concentrations (in quartiles) not associated with prognosis | Poland, Netherlands, Spain |
| Cappello 2016 [11] | 1373 pat’s w. malnutrition | 66.7 | Almost half had malignancy, 12% GI disease; increased serum B12 > 1000 pg/ml associated with increased mortality, as was recent weight loss, increased CRP, low prealbumin and impaired renal function | Italy |
| Soohoo 2017 [12] | 12968 hemodialysis pat’s | 63 | Higher serum B12 (>550 pg/ml) associated with increased mortality adjusted for age, sex, ethnicity, several comorbidities, BMI, extensive laboratory variables, appears not to be dose-dependent | USA |
| **Cardiovascular** |  |  |  |  |
| Hung 2003 [13] | 2950 participants to the 1969 survey | 48 | No relation of (low or high) serum B12 with CHD death and CVD | Australia |
| Van Oijen [14] | 211 post-CCU pat’s | 66.9 | High plasma homocysteine, but not low serum B12, increased the risk of cardiovascular morbidity and mortality in pat’s with ischemic heart disease | Netherlands |
| Dangour 2008 [15] | 853 community-dwelling adults aged >75 yrs | 78.7 | No association of serum B12 with mortality adjusted for age, sex, diabetes, history of CVD, cancer, smoking, alcohol, physical activity, homocysteine, folate | United Kingdom |
| Rafnsson [16] | 7345 participants / pat’s | 48.4 - 79.0 | Meta-analysis of 7 cohort studies: very limited evidence that vitamin B12 deficiency predisposes to the risk of mortality and morbidity from either cardiovascular diseases or diabetes in adults (meta-analysis included also [13] | USA, UK, Netherlands, Germany, Australia |
| Robinson 2011 [17] | 466 community-dwelling elderly | 75.4 | Serum B12 (quartiles) not associated with 3-yr all-cause mortality, while age, gender, and history of CVD / stroke were strongly associated | Ireland |
| Gopinath 2012 [18] | 3010 population-based participants | 67.0 | Serum B12 (quartiles) not associated with CHD and all-cause mortality adjusted for BMI, smoking, alcohol, poor self-rated health, walking disability, qualifications, renal function, presence of hypertension and/or diabetes, history of cancer or cardiovascular disease | Australia |
| Gonzalez 2007 [19] | 215 participants > 60 yrs from elderly care institutions | 75.1 | No significant association of serum B12 with mortality adjusted for age, sex, smoking habit, BMI and cognitive score | Spain |
| Mendonca 2018 [20] | 753 participants 85+ yrs |  | Higher serum B12 associated with increased all-cause and cardiovascular mortality, adjusted for sex, education, disease count, Mini-Mental State examination score, BMI, physical activity, smoking, alcohol intake; but serum B12 <170 pmol/l was taken as reference group | United Kingdom |
| Pusceddu 2019 [21] | 3316 participants undergoing coronary angiography | 63.5 | Low and high serum B12 was associated with mortality, but this association lost significance after adjustment for potential confounders. In participants with high serum B12 mortality might be related to increased inflammation. | Germany |
| Flores-Guerrero 2020 [22] | 5571 population-based albuminuria-enriched cohort | 53.5 | Positive association of higher serum B12 with all-cause mortality adjusted for age, sex, education, ethnicity, comorbidities, smoking, alcohol, history of cancer and CVD, several laboratory variables | Netherlands |

* Note: serum B12 concentrations are given either as pg/ml or pmol/l (Conversion Factor: B12 (pg/ml) x 0.738 = B12 (pmol/l)

BMI, body mass index; CHD, coronary heart disease; CVD, cardiovascular disease; ICU, Intensive Care Unit.

References

1. Baker H, Frank O, DeAngelis B. Plasma vitamin B12 titres as indicators of disease severity and mortality of patients with alcoholic hepatitis. Alcohol Alcohol 1987;22(1):1-5.

2. Salles N, Herrmann F, Sakbani K, Rapin CH, Sieber C. High vitamin B12 level: a strong predictor of mortality in elderly inpatients. J Am Geriatr Soc. 2005;53(5):917-8.

3. Salles N, Herrmann F, Sieber C, Rapin C. High vitamin B12 level and mortality in elderly inpatients. J Nutr Health Aging 2008;12(3):219-21.

4. Tal S, Shavit Y, Stern F, Malnick S. Association between vitamin B12 levels and mortality in hospitalized older adults. J Am Geriatr Soc. 2010;58(3):523-6.

5. Sviri S, Khalaila R, Daher S, Bayya A, Linton DM, Stav I, et al. Increased Vitamin B12 levels are associated with mortality in critically ill medical patients. Clin Nutr. 2012;31(1):53-9.

6. Bayir A, Ak A, Ozdinc S, Seydanoglu A, Kostekci SK, Kara F. Acute-phase vitamin B12 and folic acid levels in patients with ischemic and hemorrhagic stroke: is there a relationship with prognosis? Neurol Res. 2010;32(2):115-8.

7. Dou J, Xu W, Ye B, Zhang Y, Mao W. Serum vitamin B12 levels as indicators of disease severity and mortality of patients with acute-on-chronic liver failure. Clin Chim Acta 2012;413(23-24):1809-12.

8. Jammal M, Deneuville T, Mario N, Tiev K, Toledano C, Josselin-Mahr L, et al. [High plasmatic concentration of vitamin B12: an indicator of hepatic diseases or tumors]. Rev Med Interne 2013;34(6):337-41.

9. Callaghan FM, Leishear K, Abhyankar S, Demner-Fushman D, McDonald CJ. High vitamin B12 levels are not associated with increased mortality risk for ICU patients after adjusting for liver function: a cohort study. ESPEN J. 2014;9(2):e76-e83.

10. Van der Wal HH, Comin-Colet J, Klip IT, Enjuanes C, Grote Beverborg N, Voors AA, et al. Vitamin B12 and folate deficiency in chronic heart failure. Heart 2015;101(4):302-10.

11. Cappello S, Cereda E, Rondanelli M, Klersy C, Cameletti B, Albertini R, et al. Elevated Plasma Vitamin B12 Concentrations Are Independent Predictors of In-Hospital Mortality in Adult Patients at Nutritional Risk. Nutrients 2016;9(1):1.

12. Soohoo M, Ahmadi SF, Qader H, Streja E, Obi Y, Moradi H, et al. Association of serum vitamin B12 and folate with mortality in incident hemodialysis patients. Nephrol Dial Transplant. 2017;32(6):1024-32.

13. Hung J, Beilby JP, Knuiman MW, Divitini M. Folate and vitamin B-12 and risk of fatal cardiovascular disease: cohort study from Busselton, Western Australia. BMJ. 2003;326(7381):131.

14. van Oijen MG, Vlemmix F, Laheij RJ, Paloheimo L, Jansen JB, Verheugt FW. Hyperhomocysteinaemia and vitamin B12 deficiency: the long-term effects in cardiovascular disease. Cardiology 2007;107(1):57-62.

15. Dangour AD, Breeze E, Clarke R, Shetty PS, Uauy R, Fletcher AE. Plasma homocysteine, but not folate or vitamin B-12, predicts mortality in older people in the United Kingdom. J Nutr. 2008;138(6):1121-8.

16. Rafnsson SB, Saravanan P, Bhopal RS, Yajnik CS. Is a low blood level of vitamin B12 a cardiovascular and diabetes risk factor? A systematic review of cohort studies. Eur J Nutr. 2011;50(2):97-106.

17. Robinson DJ, O'Luanaigh C, Tehee E, O'Connell H, Hamilton F, Chin AV, et al. Vitamin B12 status, homocysteine and mortality amongst community-dwelling Irish elders. Ir J Med Sci. 2011;180(2):451-5.

18. Gopinath B, Flood VM, Rochtchina E, Thiagalingam A, Mitchell P. Serum homocysteine and folate but not vitamin B12 are predictors of CHD mortality in older adults. Eur J Prev Cardiol. 2012;19(6):1420-9.

19. Gonzalez S, Huerta JM, Fernandez S, Patterson AM, Lasheras C. Homocysteine increases the risk of mortality in elderly individuals. Br J Nutr. 2007; 97(6):1138-43.

20. Mendonca N, Jagger C, Granic A, Martin-Ruiz C, Mathers JC, Seal CJ, Hill TR. Elevated Total Homocysteine in All Participants and Plasma Vitamin B12 Concentrations in Women Are Associated With All-Cause and Cardiovascular Mortality in the Very Old: The Newcastle 85+ Study. J Gerontol A Biol Sci Med Sci. 2018;73(9):1258-64.

21. Pusceddu I, Herrmann W, Kleber ME, Scharnagl H, Marz W, Herrmann M. Telomere length, vitamin B12 and mortality in persons undergoing coronary angiography: the Ludwigshafen risk and cardiovascular health study. Aging (Albany NY) 2019;11(17):7083-97.

22. Flores-Guerrero JL, Minovic I, Groothof D, Gruppen EG, Riphagen IJ, Kootstra-Ros J, et al: Association of Plasma Concentration of Vitamin B12 With All-Cause Mortality in the General Population in the Netherlands. JAMA Netw Open 2020;3(1):e1919274.
